# Predicting Hotspots of Human-Elephant Conflict to Inform Mitigation Strategies in Xishuangbanna, Southwest China

**DOI:** 10.1371/journal.pone.0162035

**Published:** 2016-09-15

**Authors:** Ying Chen, Jorgelina Marino, Yong Chen, Qing Tao, Casey D. Sullivan, Kun Shi, David W. Macdonald

**Affiliations:** 1 Wildlife Institute, School of Nature Conservation, Beijing Forestry University, Beijing, 100083, China; 2 Wildlife Conservation Research Unit, Department of Zoology, The Recanati-Kaplan Centre, University of Oxford, Oxford, OX13 5QL, United Kingdom; 3 Xishuangbanna Forestry Administration, Yunnan, 666100, China; 4 Xishuangbanna National Nature Reserve, Yunnan, 666100, China; University of Tasmania, AUSTRALIA

## Abstract

Research on the spatial patterns of human-wildlife conflict is fundamental to understanding the mechanisms underlying it and to identifying opportunities for mitigation. In the state of Xishuangbanna, containing China’s largest tropical forest, an imbalance between nature conservation and economic development has led to increasing conflicts between humans and Asian elephants (*Elephas maximus*), as both elephant numbers and conversion of habitable land to rubber plantations have increased over the last several decades. We analyzed government data on the compensation costs of elephant-caused damage in Xishuangbanna between 2008 and 2012 to understand the spatial and temporal patterns of conflict, in terms of their occurrence, frequency and distribution. More than 18,261 incidents were reported, including episodes involving damage to rubber trees (n = 10,999), damage to crops such as paddy, upland rice, corn, bananas and sugarcane (n = 11,020), property loss (n = 689) and attacks on humans (n = 19). The conflict data reconfirmed the presence of elephants in areas which have lacked records since the late 1990s. Zero Altered Negative Binomial models revealed that the risk of damage to crops and plantations increased with proximity to protected areas, increasing distance from roads, and lower settlement density. The patterns were constant across seasons and types of crop damaged. Damage to rubber trees was essentially incidental as elephants searched for crops to eat. A predictive map of risks revealed hotspots of conflict within and around protected areas, the last refuges for elephants in the region, and along habitat corridors connecting them. Additionally, we analyzed how mitigation efforts can best diminish the risk of conflict while minimizing financial costs and adverse biological impacts. Our analytical approach can be adopted, adjusted and expanded to other areas with historical records of human-wildlife conflict.

## Introduction

Developing effective prevention and mitigation plans for human wildlife conflicts is a top conservation priority in many areas of the world [1], including China where the conversion of tropical forests to rubber plantations is threatening critical populations of the Asian elephant (*Elephas maximus*). Understanding human-elephant conflict (HEC) is important in many countries where solutions to escalating conflicts are urgently required [2–4]. In particular, knowledge of the spatial and temporal patterns of conflict can help governments and civil organizations to design more effective mitigation plans, based on reliable forecasts and maps of conflict risks.

In the State of Xishuangbanna in Southwest China, the conflicts with elephants due to damage to crops and plantations are an increasing cause of concern for both conservationists and developers. The Asian elephant is globally considered an endangered species [5] and is designated as ‘First Class National Protected Wildlife’ in China [6]. Historically widely distributed south of the Yangzi River, the growth of human populations led to their rapid withdrawal southwards [7] and they are now restricted to three states in southwest Yunnan Province [5]: Linchang, Simao, and Xishuangbanna (XSBN). Of these, XSBN contains the largest area of tropical forest and 85% of all the elephants in the country [8], concentrated in three sub-reserves: Mengyang, Mengla and Shangyong [9].

The elephant population of XSBN have been increasing over the past 30 years from an estimated 146 elephants in 1976, to 165–213 in 2006 [3], and around 250–280 at present (unpublished data). While this reflects the success of local conservation programs and favourable conservation policies, conflicts are increasing due to the economic losses from damage to crops and young rubber trees, which they frequently raid or trample. Common crops such as paddy, corn, banana, and sugarcane provide a nutritious, palatable, and easily accessible food source for wild elephants in XSBN, which are seemingly incorporating them into their foraging strategy [3, 10]. Elephants in XSBN also enter houses looking for food, resulting in property damage [8] and human deaths [11]. Presently, the insurance being paid out to farmers barely cover the costs of the damage, prompting farmers to resort to retaliatory killings [3].

The increase in HEC in XSBN is rooted in rapid land use transformations over the past 50 years [12, 13], with rubber plantations replacing traditional agriculture and forest cover, a major driver of deforestation and biodiversity loss across Southeast Asia [4]. As an irreplaceable industrial material from tropical forests, rubber plantations have been supported by the Chinese government since the 1950s and by the “Land contract responsibility system” issued in 1976, motivating local residents to grow cash crops. By the 1990’s rubber farms in XSBN were widespread, increasing by 890 km^2^ in cover between 1996 and 2005. By 2008 the plantations covered 13% of the whole state [14], while natural forests declined from 69% in 1976 to 43.6% in 2007 [15]. As the optimal environmental conditions for rubber trees and elephants are similar, plantations directly compete with elephants for living space in XSBN. With this level of habitat fragmentation and degradation, concomitant with increasing human populations (from 0.3 million in the 1950s to over a million by the 2010s in XSBN), elephants are progressively confined to protected areas (PAs). Conditions for conflict are expected to increase within and around protected areas [16]. A deeper understanding of the patterns and causes of HEC in XSBN is a prerequisite for effective mitigation planning that balances the need for economic development with Asian elephant conservation, so that priority areas can be identified where mitigation can best benefit both elephants and local communities.

Studies of patterns of crop raiding by African elephants (*Loxodonta africana*) [17–19] and Asian elephants mainly in India [20–24], have revealed some commonalities. For example, conflicts are more frequent closer to protected areas [18, 25] and roads [19], in areas with lower settlement density and close to elephant daytime refuges [17]. On the other hand, the studies have also revealed important variations at local and regional levels. Studies of HEC in China are few and typically limited to people’s attitudes and perceptions [11, 26] although the focus is now shifting towards compensation costs and prediction of localized conflicts in southern China [16, 27, 28]. We used long-term data from compensation programs in Yunnan province to provide the first systematic analysis of elephant damage in China to explore patterns and environmental correlates of conflict, including the effect of topography, land uses and other anthropogenic parameters across the entire state of XSBN and in different seasons.

Our analysis considered rubber tree and crop damage separately in order to evaluate the contributing factors to each. Additionally, we analysed the drivers of conflicts across both the rainy and dry seasons. We did not evaluate the drivers of property damage or human injury. The resulting predictive models provide specific, scientifically-based guidelines for conflict mitigation strategies, with implications for future land use policies. This study assumed no barriers to elephants travel as there are no large scale fences in the area or other infrastructure that would hamper elephant movement. While some assumptions may need to be adjusted in different areas, our analytical approach can be adopted, adjusted and expanded, to other areas with historical records of human-wildlife conflict.

## Methods

### Study area

The study area is located in Dai Autonomous Prefecture of XSBN (21° 08´-22° 36´ N, 99° 56´-101° 50´ E), Yunnan Province, in southwest China. The area borders Laos and Myanmar, and is part of the Indo-Myanmar global diversity hotspot [29]. It covers 19,582 km^2^ and supports a population of 1,133,515 people, with 39 towns in charge of 256 administrative villages (total number of settlements: 2,550). Settlements are subunits of government-defined villages and may consist of tens to hundreds of households. XSBN has a diverse cultural background, with the majority of the population (74.7%) from ethnic minorities, mainly of Dai nationality. The communities make a living cultivating paddy, alpine rice, corn and commercial plantations such as tea and coffee. Traditional activities such as collection of fruits and seasonal hunting play an important role in their life.

**Fig 1:**
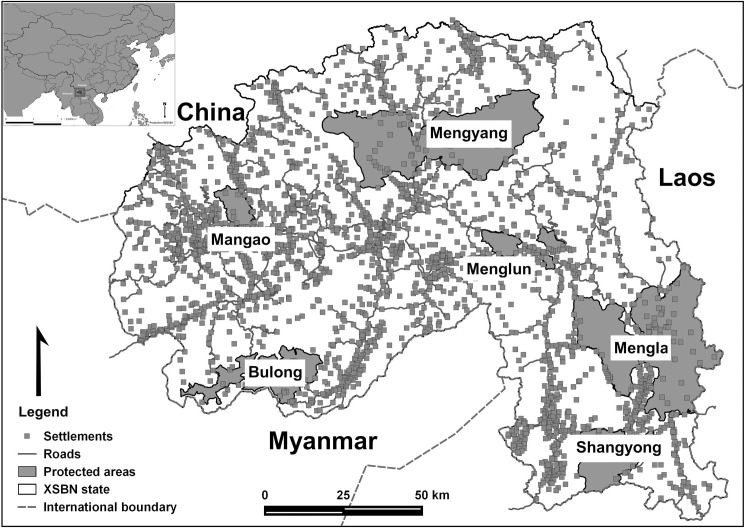
Dai Autonomous Prefecture of XSBN, southwest China. The distribution of settlements and the six sub-reserves that form the XSBN National Nature Reserve.

In the Yunnan Province, forests and elephants are protected in two national nature reserves (XSBN and Nabanhe), one state nature reserve (Bulong) and six nature reserves at city level. Altogether, they cover 19.1% of the prefecture. The largest is the XSBN National Nature Reserve (XSBN-NNR), created in 1958 and covering 2425km^2^ divided in six sub-reserves (Fig 1). This reserve harbours 20% of China’s vascular flora, 21% of the county’s mammals and 36% of the bird species [30], of which 114 are nationally protected, among them the Asian elephant and the Indochinese tiger (*Panthera tigris corbetti*).

The area has a tropical monsoon climate, with annual average temperature around 15.1–21.7 C° and 1,196–2,492 mm of annual rainfall. There is a dry season and a rainy season, which concentrates 85% of the rainfall [14]. The rainy season starts in May and the harvest period is from July to late September. Small streams dry up between November and April. Elevation ranges between 400 and 2,300 m and the area is dominated by mountains; valleys and basins occupy only 6% of the surface [31]. Nature reserves are located in mountainous area with high forest cover; settlements are concentrated at the lower elevations, in flat areas and gentle slopes. While farming and plantations are dominant livelihood activities, few physical barriers such as fences exist in the region.

### Data

We used data on human elephant conflicts collected in XSBN from 2008 to 2012. By four institutions: the Local Forest Administration, the Nature Reserves, Community Committees and, since 2010, by the insurance company “Yunnan branch, China Pacific property insurance company”, due to a change in government policy from compensation to wildlife damage insurance. At least two people in each institution are involved in reliability checks, evaluation of losses, and data storage and the collated data are submitted to the Forest Administration of Yunnan Province and yearly to the State Forestry Administration.

The data includes information on the location of the households affected, the date of the event, the species causing the problem, the type of conflict (damage to crop or damage to rubber plantation), loss assessments (area of cropland damaged, number of rubber trees damaged, number of livestock lost, number of humans attacked) and compensation amount. It is safe to assume that events of conflict were reported continuously over the study period, insofar as this is the only way for affected farmers to get compensation. The process is as follows: the household affected by wildlife immediately reports to the local reserve station, a ranger visits the site and if the damage is severe, the ranger informs the Forestry Administration. In such cases qualified staff visit the area with the ranger to record the information in the presence of one or two village leaders. Since 2010, the affected families now report to the insurance company directly, which in turn informs the Local Forest Administration.

We attributed each incident of conflict to the settlement where the affected household was located (n = 2,550 settlements). We measured candidate environmental predictors associated to each settlement within a buffer of one kilometer radius, so as to encompass the adjoining crops and plantations within the mean distance between settlements (1.16 km). We averaged values across pixels for continuous predictors, or calculated percentage of the area covered for categorical predictors (using Quantum GIS ver.2.2.0; GRASS ver.6.4.3). In order to avoid boundary problems, we calculated distances to rivers and roads by considering the neighbouring land in Laos and Burma. For settlements within protected areas we set the distance to protected area to be zero.

We postulated nine factors potentially affecting human-elephant conflict in XSBN, and standardized them using z transformations. They were settlement density [32], distance to protected area/road/river [33], elevation [34], slope [34], percentage of cropland [35], rubber tree cover, and natural forest cover [36] (S1 Table). Kendall tests confirmed that correlations amongst them were lower than 0.7 [37] (S3 Table).

### Statistical models

To explore patterns of occurrence we summarized the data as: a) count data, namely “numbers of events per settlement”; combining all events (“overall’) or separated by season and conflict type; and b) a binomial response: settlement affected by conflict (1) not affected (0). This gave us top averaged models for 1) All conflict events across both seasons, 2) all conflict events for each season (rainy/dry), 3) crop damage events, 4) rubber damage events, and 5) crop damage events for each season. Top models and full-averaged parameters are available in the S4 Table.

We created models to explain variations in occurrence and numbers of conflicts per settlement. As data showed over-dispersion, with excessive number of zeroes and large variation in positive count data (S2 Table), we fitted a zero altered negative binomial (ZANB) model [38] using the “hurdle” model in the R package pscl [39, 40]. This combines a logistic regression for the binomial response and a zero-truncated negative binomial model for the count data (only positive count data considered). Moran’s *I* test revealed spatial auto-correlation at the settlement level, (p<0.001) [41, 42], thus all models were fitted with distance-weighted autocovariates [43, 44] using the autocov_dist function in spdep [45, 46]. All data analysis was performed in R [47].

Univariate analysis was carried out for all explanatory variables, followed by multivariate analysis for significant variables (p>0.05) [42]. Univariate model residuals were tested for spatial independence using the lm.morantest function in the spdep package in R. All possible interactions were evaluated by creating a global interaction model, and significant interactions were incorporated into the multivariate analyses.

The resulting models were ranked using the Akaike Information Criterion (AIC) [48]. For each group of models, if no single model weighed over 95%, we averaged the top models (AIC<10) for each and included the averaged coefficients in the final models [49]. We adopted the Area Under the Curve (AUC) and Naglekerke R-squared methods to assess the performance of the logistic regression [50–52]. We conducted all statistical analyses in R software [47] and used the MuMIn package [49] to compare among the models’ fit. The top models for each group along with model parameters can be found in the S4 Table.

### Predictive mapping

We calculated a kernel distribution of the events of damage across the study period, and compared this against our current knowledge of elephant distribution and the location of protected areas [32]. Subsequently, we generated predictive maps of probability of occurrence of conflict by extrapolating the final averaged logistic models to the overall study area (only predictors whose coefficients’ 95% confidence intervals excluded zero were considered). To transform the logit function to a probability (p) the following formula was implemented:

$$Logit(p)=\alpha +\beta_{1}\;*\;X_{1}+\beta_{2}\;*\;X_{2}+\beta_{3}\;*\;X_{3}+\beta_{4}\;*\;X_{4}\ldots +\beta_{i}\;*\;X_{i}=A;\;p=exp(A)/(1+exp(A)),$$

where α is the intercept of the optimal model, β_i_ the X_i_ predictor’s coefficient, and p the probability of a settlement being affected by conflicts with elephants. We implemented the extrapolation with the r.mapcalculator module of Grass, QGIS [53].

## Results

### Features and distribution of elephants and conflicts in XSBN

In total, 18,261 records of conflicts with elephants were collected between 2008 and 2012 in XSBN, affecting 308 settlements. Settlements(S) experienced conflicts such as crop raiding (n_s_ = 262), damage to rubber plantations (n_s_ = 253), property loss (n_s_ = 114) and attacks on humans (n_s_ = 17). Crop raiding events (E) and damage to rubber trees were similarly common over the five year period (n_E_ = 11,020 and n_E_ = 10,999 respectively) and more frequent during the rainy season (n_E_ = 10,020 and n_E_ = 4,040 respectively). Over the study period 72% of the affected settlements suffered damages to crops and to rubber trees (n_s_ = 222). Nearly 40% of the affected settlements suffered from property loss or human attacks (S1 Fig).

**Fig 2:**
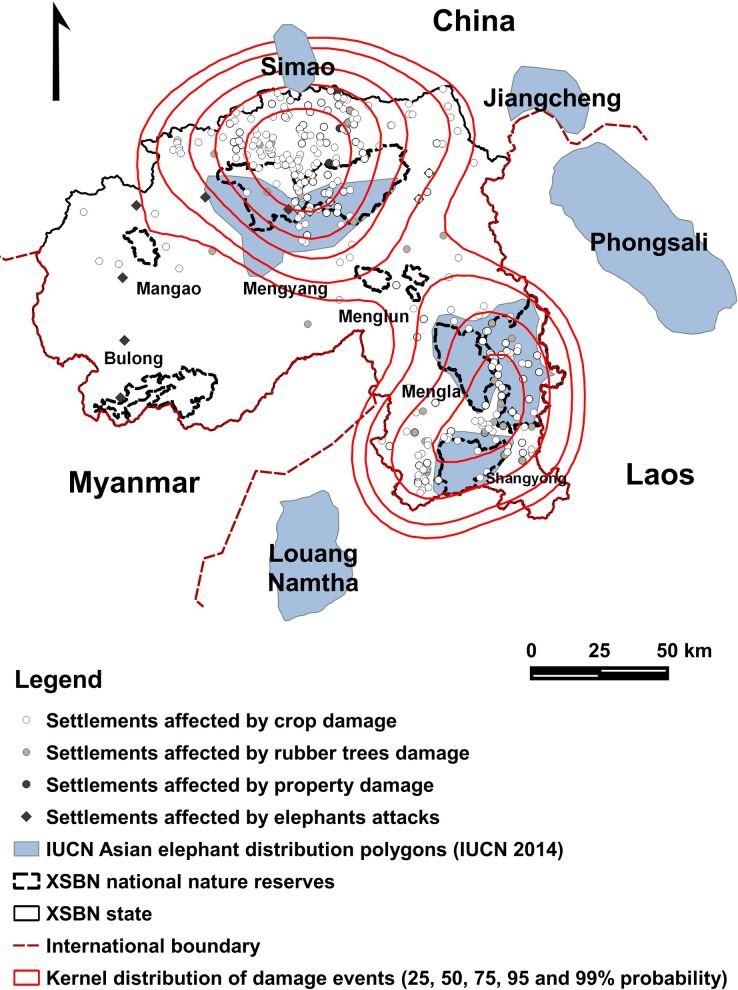
Elephant conflict kernel distributions. Kernel distributions of the events of elephant conflict per settlement in XSBN, in the context of the locations of protected areas and of elephant populations in adjacent regions.

A kernel distribution of the events of damage indicated two core areas of conflict, coincident with two sets of connected populations, namely the Simao-Mengyang system and Mengla-Shangyong system (Fig 2). Sporadic records of conflict also confirmed the presence of elephants in the sub-reserves of Mangao, Menglun and Bulong. The kernel distribution revealed a wider range than that approximated by the IUCN, with links between elephant populations within XSBN, and possibly with the neighbouring population of Simao in the Puer.

### Patterns of conflict

The spatial distribution of HEC was stable across seasons (rainy/dry) and types of conflict (all/crop/rubber). Of the nine potential predictor variables, four environmental predictors contributed to the likelihood of a settlement being affected by conflict, with relatively consistent direction and intensity across all averaged models (Table 1). The variables with most predictive power were density of settlements, and proximity to PA, both negatively correlated to the probability of conflict, and distance to roads with a positive effect. To a lesser degree, the probability was negatively associated with the area covered by rubber trees, especially in the model for all conflict events and for the rainy season models. Natural Forest cover and the interaction between proximity to PA and settlement density appeared in all of the averaged models, but neither was significant. The overall logistic model achieved the highest Nagelkerke R squared score (S5 Table), and was therefore chosen to produce the predictive map.

**Table 1:** Coefficients of significant averaged model predictors fitted to the probability of settlements being affected by elephant conflict between 2008 and 2012 years, with 95% confidence interval and notations of significance.

	All Events	By season		By type of damage		Crop Damage by Season	
		Dry	Rainy	Rubber trees	Crop events	Dry	Rainy
Distance to PA	-0.57***	-0.66***	-0.66***	-0.65***	-0.46***	-0.63***	-0.58***
	(-0.78,	(-0.96,	(-0.90,	(-0.86,	(-0.70,	(-0.93,	(-0.85,
	-0.36)	-0.36)	-0.41)	-0.44)	-0.23)	-0.34)	-0.31)
Settlement Density	-0.74***	-0.96***	-0.73***	-0.60***	-0.82***	-1.01***	-0.08***
	(-0.97,	(-1.35,	(-0.98,	(-0.83,	(-1.07,	(-1.46,	(-1.12,
	-0.51)	-0.57)	-0.48)	-0.36)	-0.57)	-0.56)	-0.56)
DRD	0.14**	0.04*	0.24***	0.21*	0.18***	—	0.24***
	(0.07,	(0.01,	(0.12,	(0.09,	(0.08,		(0.12,
	0.30)	0.30)	0.37)	0.37)	0.33)		0.37)
Rubber Tree Cover	-0.31***	—	-0.32***	-0.22*	-0.34***	—	-0.30**
	(-0.46,		(-0.49,	(-0.40,	(-0.51,		(-0.48,
	-0.15)		-0.15)	-0.03)	-0.17)		-0.12)

* P ≤ 0.05

** P ≤ 0.01

*** P ≤ 0.001

Variation in frequency of conflict among affected settlements, explained by the count data portion of the models, was best explained by settlement density, which was negatively correlated with frequency of conflict events in all models (Table 2). The interaction between settlement density and distance to PA was significant in the overall model and the rainy season models. The frequency of damage to rubber plantations was only explained by settlement density, but not by the proximity to the PA or percent rubber tree cover. Unlike the damage caused to rubber trees, the pattern of crop raiding differed between seasons—crop damage was always negatively associated to settlement density, but was also positively associated with the interaction between DPA and settlement destiny during the rainy season. Frequency was not significantly associated with proximity to the PA during the dry season not with crop cover over either season.

**Table 2:** Coefficients of significant predictors in averaged models fitted to the number of conflict events per settlement, with 95% confidence interval and notations of significance.

	All Events	By Season		By type of damage		Crop damage by season	
		Dry	Rainy	Rubber trees	Crop events	Dry	Rainy
Settlement Density	-0.48***	-0.48*	-0.34*	-0.36*	-0.50***	-0.74**	-0.30*
	(-0.75,	(-0.90,	(-0.62,	(-0.71,	(-0.77,	(-1.30,	(-0.58,
	-0.22)	-0.07)	-0.07)	-0.02)	-0.24)	-0.19)	-0.03)
DPA*Sde	0.32*	—	0.32***	—	0.32*	—	0.40**
	(0.07,		(0.07,		(0.07,		(0.13,
	0.56)		0.58)		0.57)		0.66)
Rubber Tree Cover	—	—	0.21	—	—	—	—
			(0.00,				
			0.42)				

* P ≤ 0.05

** P ≤ 0.01

*** P ≤ 0.001

### Prediction of hotspots of conflict

**Fig 3:**
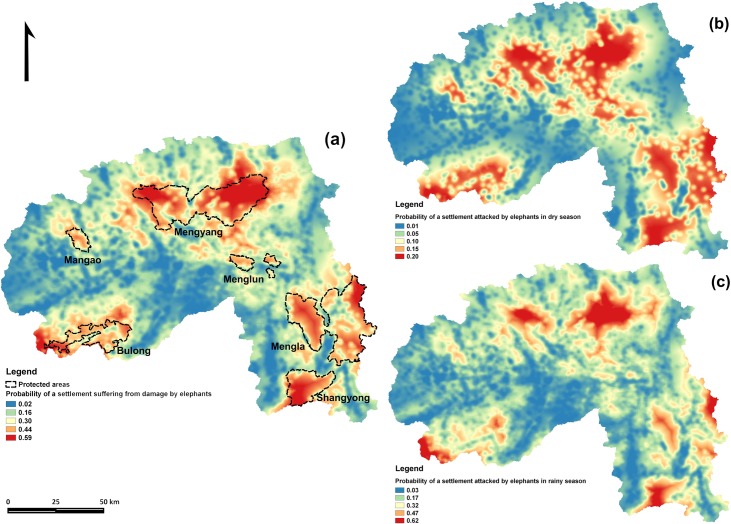
Predictive map on probability of a settlement with elephant attacks in XSBN. Overall probability across the entire state (a), and in dry (b) and rainy season (c).

The combination of factors associated to the probability of conflict revealed hotspots of conflict within PAs when extrapolated to the study area, particularly in Mengyang, Mengla and Shangyong, along the edges of PAs, along the habitat corridor connecting Mengyang and Menglun, and towards administrative boundaries close to elephant populations away from the XSBN-Puer border (Fig 3A). While the cores of conflict were similar throughout the year, the hotspots were more concentrated in the rainy season, with higher probability of experiencing conflict (21%, SD 15, versus 8%, SD 6 in dry season) (Fig 3B and 3C).

The average probability of a settlement suffering damage by elephants within the XSBN state over the study period was 23% (SD 15), over half of that predicted for settlements inside PAs (41%, SD 11). The settlements at highest risk were those inside the Mengyang sub reserve (48%, SD 12). Crops faced greater risk of damage by elephants than did rubber plantations, particularly during the rainy season, and the probability of conflict steadily decreased away from PAs. The average probability of conflict for settlements within 10km of the boundaries of Mengyang, Mengla and Shangyong sub reserves was 27% (SD 15).

## Discussion and Conclusions

Using unprecedented data, both in terms of thoroughness and temporal coverage, we unveiled critical new knowledge about patterns and drivers of human-elephant conflict in tropical forests of China, where both elephant numbers and rubber plantations are increasing. Three important findings contribute crucial information for the development of mitigation plans that might benefit both elephants and people in XSBN. Firstly, the spatial distribution of conflicts sheds light on the behavioural decisions driving crop damage and the level of connectivity between populations. Secondly, environmental and anthropogenic correlates of conflict reveal associations between land uses, level of protection and risk of conflict, both spatially and temporally. Thirdly, the resulting map of predicted risk provides detailed spatial information to guide conflict mitigation plans for HEC in XSBN.

The higher frequency of HEC incidents during the rainy season probably stems from the maturation of crops (paddy, corn, beans, peanuts, sugarcane), which are nutritious, predictable and palatable [54, 55]. There is overwhelming evidence that elephant raiding peaks near harvest time due to the ready availability of accessible food [10, 17, 22, 55, 56], despite the abundance of natural food within PAs during this time. While several factors influence the likelihood of conflict occurring, distance from the PA and its interaction with settlement density was a main driver of frequency of events during the rainy season, revealing the costs of living close to PAs, especially in small, rural settlements. Interestingly, during the dry season the main explanatory variable of frequency of conflict was settlement density, regardless of distance to PA. This may mean that during the dry season food in the PA is scarce and elephants are willing to travel further to find food, though they still avoid dense settlements.

The hotspots of conflict centred on Simao-Mengyang and Mengla-Shangyong connecting zones, and subscribe to the “Double Core Structure” (DCS) pattern, with two major zones or ‘cores’ of conflict within XSBN (Fig 2). The DCS pattern in XSBN was mainly driven by human dominated factors—settlement density, proximity to PAs and distance to roads. The impact of other topographical or land use factors was weakened in the complex anthropogenic landscape. Because natural food diminishes quickly beyond PAs due to the expanding and deepening human footprint (dense settlements, increasing farmland and cash crops, infrastructure, deforestation), elephants tend to forage further away into cropland. With expanding deforestation, the crops closer to PAs bear the brunt of damage. Cropland near PAs was at the highest risk of raiding, but elephants would sometimes travel up to 30km to raid crops in XSBN. Similarly, Gubbi (2012) determined that the highest incidence of conflict with elephants was 3.1–5.0 km away, and up to 10km, from the 5,000 km^2^ Nagarahole National Park in Southern India.

Perhaps surprisingly, area of crop cover did not appear as a significant variable in any of the models. It seems to follow that in areas where crop raiding is a frequent event, crop cover would be a dominant driver. However, it appears that instead elephants raid crops that are close to the PAs, far from roads, and have low settlement density opportunistically, as opposed to searching for areas with higher crop coverage. Larger crop cover may be found near more densely populated settlements, which generally occur further from the PA and closer to roads, decreasing the likelihood of an elephant raid regardless of crop cover. Conflict will always be high at the edges of PAs, but the greater the area of natural habitat in PAs, corridors or restored land, the less likely it is that elephants would wander into human-dominated landscapes

Sightings of elephants near the Laos border, and between Simao and Mengyang [14], and documented migration between XSBN and Jiangcheng [57] all indicate that local elephant populations in XSBN may work as a metapopulation linked to Puer, Jiangcheng, and Laos (Loung, Namthe, and Phongsali) [5]. The conflict data also revealed the presence of elephants around Menglun, Bulong, and Mangao reserves, with no record of a permanent population since the 1990s [58]. The reports of elephant attacks around Menglun, lacking a permanent elephant population, also indicate a potential connection between Mengla and Mengyang populations, with Menglun as an intermediate point (Fig 2). While these protected areas may serve only as temporary refuges for elephants searching for crops, they provide opportunities for gene flow among the resident sub-populations. For these discrete stepping stones to be insufficient to ensure dispersal within the metapopulation, future conservation measures in XSBN should address the entire metapopulation to ensure long-term viability.

The fragmented PAs of XSBN might be too small to sustain healthy elephant population, but habitat restoration in the form of artificial corridors can allow more dispersal and gene flow. The prompt creation of dispersal corridors before current channels are entirely blocked must be a priority. Among land use policies that can enhance elephant habitats are the temporal burning of forest in PAs to promote the regeneration of wild plants and supplementing natural food to diminish the need for elephants to leave natural habitats and HEC outside of the PAs.

A major component of HEC in XSBN is the damage caused to rubber plantation. While elephants occasionally eat tender leaves and bark to make up for mineral deficiencies in their diet [16], the damage they cause to trees mostly occurs on their route searching for more palatable crops, and thus is likely incidental [3, 55]. In India, for example, inedible cotton is damaged as elephants look for food [22]. Our data also support this behavioural pattern, as damage to crops and rubber trees were largely parallel in XSBN, and increasing rubber tree cover was significantly negatively correlated with both crop and rubber damage, indicating that elephants are not seeking out rubber trees, and may even be avoiding them.

Our study shares the limitations faced by other attempts to predict hotspot of conflict, rooted in our knowledge gap of the behaviour, ecology, and status of Asian elephants in XSBN. There are inherent problems with using predictive conflict hotspot maps to advise mitigation activities. The hotspots will change depending on development, restoration efforts, population change, and as a result of conflict mitigation measures. Therefore, it is necessary to better understand why elephants are leaving the PA. While we studied HEC patterns and its driving mechanism in depth, further studies will be necessary to comprehend the behaviour of elephants in XSBN and other regions with similar land use patterns: Do they leave protected areas because food in their natural habitats is not abundant? Do they risk encountering humans raiding crops simply because crops are highly predictable, palatable, abundant and easy accessible? As deforestation and encroachment continue to limit natural habitat, the elephants compressed within PAs will continue to roam into the human dominated landscape search for food, increasing the conflict with humans. Resettling people might be unfeasible for local governments in XSBN, and local residents may not be able bear the economic costs of crops raiding or property damage for much longer. In this scenario, future measurements to mitigate conflict should prioritize the hotspots of conflicts identified in this study, with a comprehensive Asian elephant conservation plan that takes into account the elephant’s metapopulation structure in XSBN.

Conflict mitigation actions in XSBN should prioritize settlements in the double core of connecting zones, particularly during the rainy season. The analytical approach that we present can be adopted for other areas with historical records of human-wildlife conflict, but to improve the predictive value of spatial models we advise the inclusion of more precise information on the location of the crops or rubber trees damaged (in our dataset, the location of a conflict is that of the settlement); and ongoing mitigation efforts; and the integration of information on elephant population status and dynamics. In our case, there were no large scale mitigation trials undertaken during the study period. There have been scattered elephant population surveys in Mengyang, Shangyong, and Mengla sub reserves since 2003 [59–61], but information on population size generated from the diverse survey methods during different study periods is difficult to reconcile.

The conversion of land to rubber plantations directly translates into loss of habitat for elephants. In 2010 50% of all rubber production in China came from XSBN, with the plantations covering 6,000 km^2^ and involving 95% of the settlements [14]. Stopping new plantations might sound unrealistic, but supply currently exceeds the global demand for rubber and rubber prices reached a five year low in 2011 [62]. Any effort to stop or reduce the area covered by rubber trees should focus on the edges of protected areas, particularly areas with low human density, elephant corridors, and to avoid areas adjoining forest blocks or farmland in order to decrease incidental damage. Eco-compensation for households reverting cropland/plantations of low commercial value to forests is one way to promote such land use policies.

## Supporting Information

S1 Fig Number of settlements affected by elephant according to conflict type and their combinations (damage to crops, rubbers, property and attack on human in short “Crop” “Rubber” “Property” and “Human”). (DOCX)

S1 Table Description of the environmental variables postulated as predictors of human-elephant conflict in XSBN. (DOCX)

S2 Table Descriptive statistics (variance and mean) of the response variables: number of conflict events and number of settlements affected. (DOCX)

S3 Table Correlation of Environmental Predictor Variables. (DOCX)

S4 Table AIC top models and averaged weights for each model group, along with Log Likelihood delta AIC values. (DOCX)

S5 Table Models’ performance measured as AUC scores and Nagelkerke R-squared. (DOCX)

S6 Table a) Coefficients of averaged model variables influencing the probability of a settlement suffering damage by elephant with 95% CI and indices of significance. b) Coefficients of averaged model variables influencing number of event occurrences in a settlement suffering damage by elephants with 95% CI and indices of significance.
(DOCX)
