# Supplementary material for: Predicting Hotspots of Human-Elephant Conflict to Inform Mitigation Strategies in Xishuangbanna, Southwest China
Source: PLoS One. 2016 Sep 15;11(9):e0162035. doi: 10.1371/journal.pone.0162035 (PMC5025021; doi:10.1371/journal.pone.0162035)
Supplement: S1 Fig — (DOCX) [file pone.0162035.s001.docx]

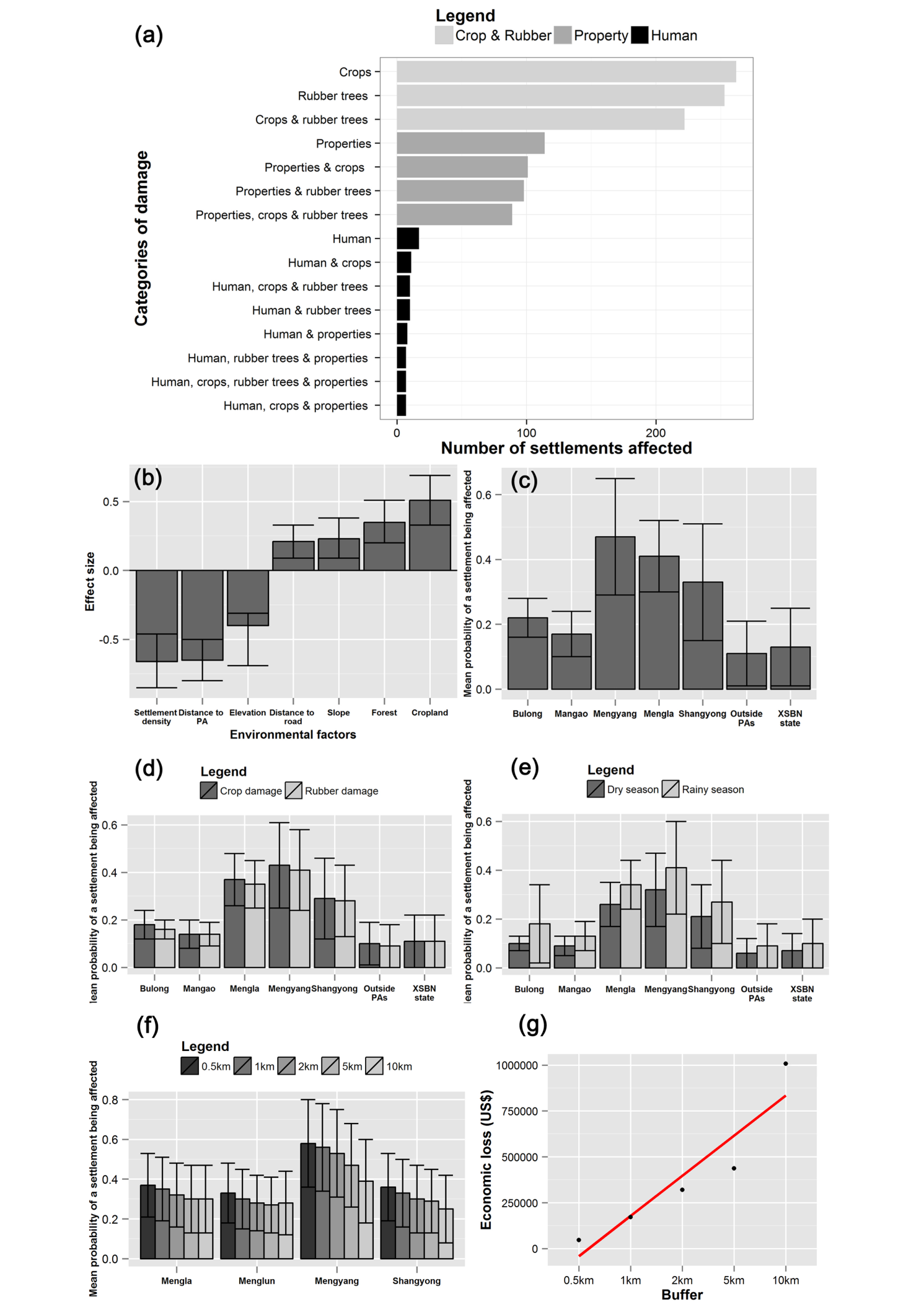


Figure S1. Number of settlements affected by elephant according to conflict type and their combinations (damage to crops, rubbers, property and attack on human in short “Crop” “Rubber” “Property” and “Human”)
