# Supplementary material for: Predicting Hotspots of Human-Elephant Conflict to Inform Mitigation Strategies in Xishuangbanna, Southwest China
Source: PLoS One. 2016 Sep 15;11(9):e0162035. doi: 10.1371/journal.pone.0162035 (PMC5025021; doi:10.1371/journal.pone.0162035)
Supplement: S1 Table — (DOCX) [file pone.0162035.s002.docx]

**Supporting Information**

Table S1. Description of the environmental variables postulated as predictors of human-elephant conflict in XSBN.

| **Categories** | **Covariates** | **Code** | **Details** |
| --- | --- | --- | --- |
| Anthropogenic | Distance to protected area (km) | DPA | Distance from settlement to closest protected area (Source: XSBN National Nature Reserve) |
|  | Distance to road (km) | DRD | Distance from settlement to road (all types of roads) (Source: XSBN National Nature Reserve) |
|  | Settlement density (per 100km^2^) | Sde | Density of settlements (kernel) (OkabeSatoh and Sugihara, 2009) |
| Topographical | Distance to river (km) | DR | Distance from settlement to permanent river (Source: http://freegisdata.rtwilson.com/) |
|  | Elevation(km) | Elev | Elevation from STRM 30m Digital Elevation Map (Source: http://glcf.umd.edu/data/srtm/) |
|  | Slope (degree) | Slop | Slope from STRM 30m Digital Elevation Map (Source: <http://glcf.umd.edu/data/srtm/>) |
| Land use | Cropland (% cover) | Crop | Percentage cover from land cover data in 2009 (Source: <http://data.ess.tsinghua.edu.cn>; resolution: 30m) |
|  | Natural forest (% cover) | Forest | Percentage cover from satellite data, 2010 (Source: (SenfPflugmachervan der Linden *et al.*, 2013), resolution: 250m) |
|  | Rubber plantations(% cover) | Rubber | Percentage cover from satellite data, 2010 (Source: (SenfPflugmachervan der Linden *et al.*, 2013); resolution: 250m) |
