# Supplementary material for: Predicting Hotspots of Human-Elephant Conflict to Inform Mitigation Strategies in Xishuangbanna, Southwest China
Source: PLoS One. 2016 Sep 15;11(9):e0162035. doi: 10.1371/journal.pone.0162035 (PMC5025021; doi:10.1371/journal.pone.0162035)
Supplement: S2 Table — (DOCX) [file pone.0162035.s003.docx]

Table S2. Descriptive statistics (variance and mean) of the response variables: number of conflict events and number of settlements affected.

| **Response** | **Mean** | **Variance** | **Total Events** | **Total Settlements** |
| --- | --- | --- | --- | --- |
| All events | 7.16 | 1428.53 | 18261 | 308 |
| Dry season | 1.58 | 123.12 | 4040 | 153 |
| Rainy season | 3.93 | 465.44 | 10020 | 248 |
| Rubber tree damage | 4.31 | 720.40 | 10999 | 253 |
| Crop damage | 4.32 | 518.21 | 11020 | 262 |
| Crop damage, dry season | 0.75 | 36.29 | 1914 | 125 |
| Crop damage, rainy season | 2.59 | 193.16 | 6617 | 223 |
