# Supplementary material for: Predicting Hotspots of Human-Elephant Conflict to Inform Mitigation Strategies in Xishuangbanna, Southwest China
Source: PLoS One. 2016 Sep 15;11(9):e0162035. doi: 10.1371/journal.pone.0162035 (PMC5025021; doi:10.1371/journal.pone.0162035)
Supplement: S3 Table — (DOCX) [file pone.0162035.s004.docx]

Table S3. Correlation of environmental predictor variables

|  | DPA | DRD | DR | Elevation | Slope | Sde | Crop | Rubber | Forest |
| --- | --- | --- | --- | --- | --- | --- | --- | --- | --- |
| DPA |  |  |  |  |  |  |  |  |  |
| DRD | 0.01 |  |  |  |  |  |  |  |  |
| DR | 0.08 | 0.18 |  |  |  |  |  |  |  |
| Elevation | 0.15 | 0.14 | 0.32 |  |  |  |  |  |  |
| Slope | 0.09 | 0.01 | 0.07 | 0.19 |  |  |  |  |  |
| Sde | 0.04 | -0.40 | -0.17 | -0.16 | -0.03 |  |  |  |  |
| Crop | -0.05 | -0.22 | -0.17 | -0.16 | 0.13 | 0.41 |  |  |  |
| Rubber | -0.04 | -0.06 | -0.09 | -0.56 | -0.24 | -0.04 | -0.44 |  |  |
| Forest | 0.03 | 0.35 | 0.17 | 0.47 | 0.06 | -0.36 | -0.46 | -0.33 |  |
