# Supplementary material for: Predicting Hotspots of Human-Elephant Conflict to Inform Mitigation Strategies in Xishuangbanna, Southwest China
Source: PLoS One. 2016 Sep 15;11(9):e0162035. doi: 10.1371/journal.pone.0162035 (PMC5025021; doi:10.1371/journal.pone.0162035)
Supplement: S4 Table — (DOCX) [file pone.0162035.s005.docx]

Table S4. AIC top models and averaged weights for each model group, along with Log Likelihood delta AIC values.

| **Model Variables** | **Log Likelihood** | **∆ AIC** | **Weight** |
| --- | --- | --- | --- |
| **All Conflict Events** | | | |
| DPA*Sde+DRD+Forest+Rubber+AC | -2226.10 | 0.00 | 0.74 |
| DPA*Sde+Rubber+AC | -2231.72 | 3.13 | 0.15 |
| DPA*Sde+Rubber+Slop+AC | -2230.30 | 4.33 | 0.08 |
| DPA*Sde+Forest+Rubber+AC | -2231.47 | 6.88 | 0.02 |
| **Crop Damage Events** | | | |
| DPA*Sde+DRD+Forest+Rubber+AC | -1863.76 | 0.00 | 0.88 |
| DPA*Sde+Rubber+Slop+AC | -1868.53 | 5.49 | 0.06 |
| DPA*Sde+Rubber+AC | -1870.67 | 5.72 | 0.05 |
| DPA*Sde+Forest+Rubber+AC | -1870.30 | 9.03 | 0.01 |
| **Rubber Damage Events** | | | |
| DPA*Sde+DRD+Forest+Rubber+AC | -1773.93 | 0.00 | 0.27 |
| DPA*Sde+Rubber+AC | -1774.20 | 0.55 | 0.20 |
| DPA*Sde+Forest+Rubber+AC | -1772.56 | 1.32 | 0.14 |
| DPA*Sde+Rubber+Slop+AC | -1772.98 | 2.17 | 0.09 |
| DPA+DRD+Forest+Rubber+Sde+AC | -1777.13 | 2.35 | 0.08 |
| DPA*DRD+Forest+Rubber*Sde+AC | -1777.27 | 2.74 | 0.07 |
| DPA*Sde+DRD+Forest*Rubber+AC | -1773.27 | 2.75 | 0.07 |
| DPA*Rubber+Sde+AC | -1779.63 | 3.31 | 0.05 |
| DPA+DRD+Forest+Rubber+Sde+AC | -1775.83 | 3.81 | 0.04 |
| **Dry Season All Conflict Events** | | | |
| DPA*Sde+Slop+AC | -1080.90 | 0.00 | 0.30 |
| DPA+Forest*Sde+AC | -1081.27 | 0.73 | 0.21 |
| DPA+DRD+Forest*Sde+AC | -1079.30 | 0.84 | 0.20 |
| DPA*Sde+Rubber+AC | -1081.61 | 1.42 | 0.15 |
| DPA*Sde+Rubber+Slop+AC | -1080.16 | 2.56 | 0.08 |
| DPA*Sde+DRD+Forest+Rubber+AC | -1078.98 | 4.25 | 0.04 |
| DPA*Sde+Forest+Rubber+AC | -1081.51 | 5.26 | 0.02 |
| DPA*Sde+Forest*Rubber+Slop+AC | -1079.01 | 8.37 | 0.00 |
| **Rainy Season All Conflict Events** | | | |
| DPA*Sde+DRD+Forest+Rubber+AC | -1711.68 | 0.00 | 1.0 |
| **Dry Season Crop Damage Events** | | | |
| DPA+Forest*Sde+AC | -830.26 | 0.00 | 0.42 |
| DPA+DRD+Forest*Sde+AC | -828.49 | 0.50 | 0.33 |
| DPA*Sde+Slop+AC | -831.25 | 1.98 | 0.16 |
| DPA*Sde+Rubber+Slop+AC | -830.62 | 4.75 | 0.04 |
| DPA*Sde+Rubber+AC | -832.85 | 5.7 | 0.03 |
| DPA*Sde+Forest*Rubber+Slop+AC | -828.05 | 7.74 | 0.01 |
| DPA*Sde+Forest+Rubber+AC | -832.32 | 8.16 | 0.01 |
| DPA*Sde+DRD+Forest+Rubber+AC | -830.41 | 8.40 | 0.01 |
| **Rainy Season Crop Damage Events** | | | |
| DPA*Sde+DRD+Forest+Rubber+AC | -1549.03 | 0.00 | 1.00 |
