# Supplementary material for: Predicting Hotspots of Human-Elephant Conflict to Inform Mitigation Strategies in Xishuangbanna, Southwest China
Source: PLoS One. 2016 Sep 15;11(9):e0162035. doi: 10.1371/journal.pone.0162035 (PMC5025021; doi:10.1371/journal.pone.0162035)
Supplement: S5 Table — (DOCX) [file pone.0162035.s006.docx]

Table S5. Models’ performance measured as AUC scores and Nagelkerke R-squared.

|  | All events | By season | | By type of damage | | Crop damage by season | |
| --- | --- | --- | --- | --- | --- | --- | --- |
|  |  | Dry | Rainy | Crop | Rubber Trees | Dry | Rainy |
| Nagelkerke R squared | 0.26 | 0.17 | 0.16 | 0.26 | 0.26 | 0.16 | 0.16 |
| AUC | 0.81 | 0.76 | 0.76 | 0.78 | 0.81 | 0.79 | 0.77 |
| Sensitivity | 0.75 | 0.86 | 0.95 | 0.76 | 0.41 | 0.74 | 0.81 |
| Specificity | 0.73 | 0.57 | 0.86 | 0.72 | 0.96 | 0.73 | 0.62 |
