# Supplementary material for: Predicting Hotspots of Human-Elephant Conflict to Inform Mitigation Strategies in Xishuangbanna, Southwest China
Source: PLoS One. 2016 Sep 15;11(9):e0162035. doi: 10.1371/journal.pone.0162035 (PMC5025021; doi:10.1371/journal.pone.0162035)
Supplement: S6 Table — a) Coefficients of averaged model variables influencing the probability of a settlement suffering damage by elephant with 95% CI and indices of significance. b) Coefficients of averaged model variables influencing number of event occurrences in a settlement suffering damage by elephants with 95% CI and indices of significance. (DOCX) [file pone.0162035.s007.docx]

Table S6. a) Coefficients of averaged model variables influencing the probability of a settlement suffering damage by elephant with 95% CI and indices of significance.

|  | **All Events** | **Rainy Season Events** | **Dry Season Events** | **All Crop Events** | **Rainy Season Crop Events** | **Dry Season Crop Events** | **Rubber**  **Damage Events** |
| --- | --- | --- | --- | --- | --- | --- | --- |
| **DPA** | -0.569*** | -0.657*** | -0.662*** | -0.464*** | -0.578*** | -0.631*** | -0.647*** |
|  | (-0.783, | (-0.904, | (-0.961, | (-0.701, | (-0.849, | (-0.926, | (-0.856, |
|  | -0.355) | -0.409) | -0.363) | -0.226) | -0.307) | -0.337) | -0.439) |
| **Sde** | -0.739*** | -0.727*** | -0.961*** | -0.819*** | -0.0840*** | -1.008*** | -0.596*** |
|  | (-0.970, | (-0.978, | (-1.349, | (-1.073, | (-1.116, | (-1.461, | (-0.834, |
|  | -0.508) | -0.475) | -0.574) | -0.565) | -0.564) | -0.555) | -0.358) |
| **DRD** | 0.137** | 0.244*** | 0.036* | 0.180*** | 0.241*** | 0.049 | 0.214* |
|  | (0.067, | (0.121, | (0.007, | (0.083, | (0.116, | (-0.011, | (0.087, |
|  | 0.303) | 0.367) | 0.299) | 0.325) | 0.366) | 0.302) | 0.365) |
| **Forest Cover** | -0.037 | -0.057 | -0.161 | -0.046 | -0.051 | -0.283 | -0.086 |
|  | (-0.186, | (-0.204, | (-0.939, | (-0.195, | (-0.203, | (-0.992, | (-0.285, |
|  | 0.088) | 0.089) | 0.250) | 0.912) | 0.101) | 0.258) | 0.104) |
| **Rubber Tree Cover** | -0.308*** | -0.321*** | -0.043 | -0.340*** | -0.302** | -0.013 | -0.217* |
|  | (-0.463, | (-0.493, | (-0.351, | (-0.511, | (-0.484, | (-0.372, | (-0.401, |
|  | -0.153) | -0.150) | 0.060) | -0.169) | -0.120) | 0.100) | -0.034) |
| **Slop** | 0.011 | -- | 0.076 | 0.010 | -- | 0.054 | -- |
|  | (-0.022, |  | (-0.010, | (0.007, |  | (0.037, |  |
|  | 0.282) |  | 0.402) | 0.336) |  | 0.492) |  |
| **DPA*Sde** | -0.017 | -0.145 | 0.017 | 0.042 | -0.106 | 0.036 | 0.023 |
|  | (-0.255, | (-0.417, | (-0.349, | (-0.219, | (-0.401, | (-0.257, | (-0.195, |
|  | 0.221) | 0.127) | 0.405) | 0.304) | 0.190) | 0.545) | 0.325) |
| **Forest*Sde** | -- | -- | -0.182 | -- | -- | -0.325 | -- |
|  |  |  | (-0.959, |  |  | (-0.989, |  |
|  |  |  | 0.062) |  |  | 0.123) |  |
| **Forest*Rubber** | -- | -- | 0.314e-3 | -- | -- | 0.001 | -0.010 |
|  |  |  | (-0.193, |  |  | (-0.196, | (-0.068, |
|  |  |  | 0.330) |  |  | 0.376) | 0.381) |
| **AC** | 0.006*** | 0.005*** | 0.005*** | 0.006*** | 0.006*** | 0.005*** | 0.006*** |
|  | (0.005, | (0.004, | (0.004, | (0.005, | (0.005, | (0.004, | (0.005, |
|  | 0.007) | 0.006) | 0.006) | 0.007) | 0.007) | 0.006) | 0.007) |

* P ≤ 0.05, ** P ≤ 0.01, *** P ≤ 0.001

b) Coefficients of averaged model variables influencing number of event occurrences in a settlement suffering damage by elephants with 95% CI and indices of significance.

|  | **All Events** | **Rainy Season Events** | **Dry Season Events** | **All Crop Events** | **Rainy Season Crop Events** | **Dry Season Crop Events** | **Rubber**  **Damage Events** |
| --- | --- | --- | --- | --- | --- | --- | --- |
| **DPA** | -0.037 | 0.029 | 0.157 | 0.008 | 0.115 | 0.191 | -0.216 |
|  | (-0.296, | (-0.239, | (-0.218, | (-0.253, | (-0.163, | (-0.235, | (-0.570, |
|  | 0.222) | 0.296) | 0.531) | 0.269) | 0.392) | 0.617) | 0.137) |
| **Sde** | -0.484*** | -0.341* | -0.482* | -0.500*** | -0.302* | -0.743** | -0.361* |
|  | (-0.752, | (-0.615, | (-0.897, | (-0.765, | (-0.578, | (-1.296, | (-0.705, |
|  | -0.216) | -0.067) | -0.067) | -0.235) | -0.026) | -0.191) | -0.017) |
| **DRD** | 0.057 | 0.059 | 0.949e-3 | 0.079 | 0.084 | 0.022 | 0.008 |
|  | (-0.048, | (-0.050, | (-0.171, | (-0.028, | (-0.026, | (-0.150, | (-0.138, |
|  | 0.204) | 0.169) | 0.179) | 0.207) | 0.053) | 0.279) | 0.154) |
| **Forest Cover** | -0.028 | -0.126 | -0.115 | -0.049 | -0.103 | 0.334 | -0.120 |
|  | (-0.216, | (-0.287, | (-0.383, | (-0.217, | (-0.259, | (-0.281, | (-0.150, |
|  | 0.143) | 0.034) | 0.874) | 0.108) | 0.194) | 1.146) | 0.404) |
| **Rubber Tree Cover** | 0.126 | 0.209 | -0.007 | 0.006 | 0.086 | -0.015 | 0.210 |
|  | (-0.092, | (0.002, | (-0.279, | (-0.196, | (-0.113, | (-0.570, | (0.126, |
|  | 0.344) | 0.415) | 0.324) | 0.208) | 0.284) | 0.245) | 0.545) |
| **Slop** | 0.001 | -- | 0.028 | -0.001 | -- | 0.027 | -- |
|  | (-0.198, |  | (-0.230, | (-0.207, |  | (-0.248, |  |
|  | 0.213) |  | 0.375) | 0.186) |  | 0.508) |  |
| **DPA*Sde** | 0.316* | 0.321*** | 0.182 | 0.319* | 0.397** | 0.026 | 0.118 |
|  | (0.073, | (0.066, | (-0.029, | (0.071, | (0.132, | (-0.355, | (0.041, |
|  | 0.558) | 0.576) | 0.641) | 0.567) | 0.663) | 0.543) | 0.617) |
| **Forest*Sde** | -- | -- | 0.145 | -- | -- | 0.459 | -- |
|  |  |  | (-0.195, |  |  | (0.011, |  |
|  |  |  | 0.911) |  |  | 1.215) |  |
| **Forest*Rubber** | -- | -- | -0.001 | -- | -- | -0.005 | 0.001 |
|  |  |  | (-0.753, |  |  | (-1.27, | (-0.336, |
|  |  |  | 0.147) |  |  | 0.022) | 0.368) |
| **AC** | 0.003** | 0.002*** | 0.002*** | 0.002*** | 0.002*** | 0.001* | 0.003*** |
|  | (0.002, | (0.002, | (0.001, | (0.001, | (0.001, | (0.000, | (0.002, |
|  | 0.004) | 0.003) | 0.003) | 0.003) | 0.003) | 0.003) | 0.004) |

* P ≤ 0.05, ** P ≤ 0.01, *** P ≤ 0.001
